# Immunological Testing Reveals Exposure to Malaria in the Hypoendemic Region of Iran

**DOI:** 10.1155/2014/614287

**Published:** 2014-10-09

**Authors:** Narges Obeidi, G-Halli Rajasekariah, Iraj Nabipour, Roya Amirinejad, Diane Dogcio, Habib Emami

**Affiliations:** ^1^The Persian Gulf Tropical Medicine Research Center, Bushehr University of Medical Sciences, Bushehr, Iran; ^2^Cellabs Pty Ltd, Unit 7, No. 27 Dale Street, Brookvale, NSW 2100, Australia; ^3^Department of Epidemiology & Biostatistics, National Research Institute of Tuberculosis and Lung Disease (NRITLD), Tehran, Iran

## Abstract

*Background*. South eastern parts of Iran remain endemic for malaria infection. There is some concern that malaria infection may spread into Bushehr, which is located in the south western part bordering the Persian Gulf and at the periphery of the declared endemic region Hormozgan province due to frequency of visitors from eastern endemic areas and from neighboring malaria endemic countries. We investigated malaria prevalence in Bushehr. *Methods and Results*. Attempts were made to identify malaria active infection in blood smears and malaria specific antibody and antigens in serum samples. Traditional blood smears prepared from 1955 blood specimens yielded no definitive malaria positive case by microscopic technique. A total of 270 (13.8%) serum samples were positive for malaria antibodies. Using specific ELISA kits, presence of histidine rich proteins and lactate dehydrogenase antigens were investigated in serum samples. No histidine rich proteins specific for *P. falciparum* were detected amongst 270 antibody positive samples. However, six samples representing 0.3% of total population, were found to be positive for plasmodium pan specific lactate dehydrogenase antigens. This suggested the possibility of low level exposure to malaria in Bushehr community. *Conclusions*. Out of a total of 1955 samples tested, 270 (13.8%) were positive for malaria antibodies and six (0.3%) of these were positive for plasmodium-specific lactate dehydrogenase antigen suggesting a low level exposure to malaria in a hypoendemic region based on immunological testing. Since none of the 270 antibody samples were positive for histidine rich protein antigens, there is scope for further testing of blood samples by molecular methods such as polymerase chain reactions to confirm the plasmodium species and provide information valuable for future investigations. Our testing strategy for hypoemdemic malaria can be used as a template for investing malaria in 32 eliminating countries for testing ongoing transmission. This approach may be useful as a method in epidemiological studies.

## 1. Background

Several publications show importance of malaria control and elimination programs for economic progress [1]. In Iran over the years, in association with WHO (Roll Back Malaria) and UNDP programs, considerable amount of effort has been expended by health authorities to control and prevent malaria through various measures including residual house spraying, larvicidal treatment, biological control measures and community education in using bed nets, and provision of antimalaria drug therapy [2, 3]. Malaria in Iran has been recently reviewed [4, 5]. Three provinces (Kerman, Hormozgan, and Sistan-Baluchistan) recorded 16,000 during 2005 to 2007 and following the control measures the incidence were reduced to just 2166 cases in 2010 [3]. These provinces share common borders with Afghanistan and Pakistan where malaria is endemic. Visitors from neighboring endemic countries come and work temporarily in Iran and they may bring in infection into new foci. We investigated in some detail such a possibility in the Bushehr province, which is located interior to the endemic region and where foreign workers have access. Investigation was done where external workers from neighboring endemic regions and work in Bushehr province. Our strategy involved using both traditional methods and novel immunological assays to assess if malaria was prevalent or not. If prevalent, then the exposed individuals should show some immunological response to malaria exposure and at least in the induction of malaria specific antibodies [6]. Those exposed individuals showing proven level of malaria antibodies were further tested for the presence of malaria antigens in the serum samples. Presence of malaria antigen is suggestive of malaria exposure in the community. This approach was proved to be useful in identifying people exposed to malaria.

## 2. Methods

### 2.1. Blood Samples

**Figure 1:**
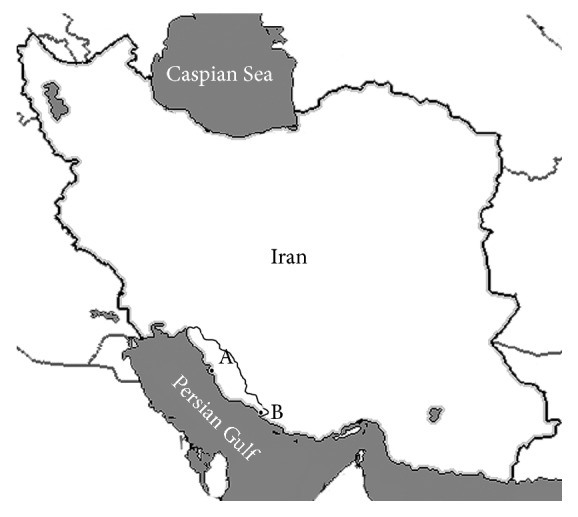
Map of Iran and the region where malaria work is undertaken (marked A and B in the figure). Area B shows the location of Gaz Refinery where samples were collected. Samples were transported to area A and tested in this area.

Informed consent was obtained from all participants and this project was approved by The Persian Gulf Tropical Medicine Research Center, Bushehr University of Medical Sciences. Blood samples used in this study were provided by voluntary individuals who willingly participated in this study of malaria investigation. The health status of voluntary blood donors met the required condition as stipulated by the Ethical committee for using their blood samples in this investigation. Of the sample donors, none was suffering from any symptoms suggestive of malaria, none had ever been hospitalised with malaria. The region where work was undertaken is marked on the map (Figure 1). Region A is Bushehr, which is an urban residential locality. Area B is Asalooyeh which contains both residential and industrial localities. The Gaz Refinery located in Asalooyeh attracts several guest workers (designated “travelers”) from neighboring endemic regions as well as adjacent endemic countries. Samples from area B were tested in view of the good mix of guest workers coming into work at Gaz Refinery as well as the local residents in area B. The plan of study as well as the objective of this study in identifying any potential cases of malaria exposure and/or malaria carriers was explained to the participants. Altogether a total of 1955 blood samples were obtained from ten geographically different centers (Table 1). Specimens were collected during a five-month period from August to December 2009. One mL blood samples were collected into K2-EDTA (ethylenediaminetetraacetic acid dipotassium salt) tubes for microscopy and other tests. A second 4 mL blood sample was collected in plain tubes and serum was separated by clotting. Collected specimens were frozen and stored at –20°C until tested. Blood films were prepared from specimens in EDTA tubes.

### 2.2. Blood Smear Microscopic Examination

Thick and thin blood smears were prepared based on the traditional methods described in the Medical Parasitology manual and other publications [7, 8]. The blood smears were stained with Giemsa (MERCK, Germany) and slides were examined under oil immersion by trained technicians using Olympus Research Microscope (Cx21). Slides showing proven blood stages of* P. falciparum* and* P. vivax* parasites were used as reference during malaria microscopy. For* P. malariae* and* P. ovale*, slides were not available, therefore published pictures were used as a reference. Cross-checking of slides to confirm diagnosis was also performed between technicians.

### 2.3. Immunoassay for Malaria Antibody

Malaria specific antibodies present in the serum samples were detected by using a commercially available ELISA kit (PAN Malaria Antibody CELISA, Cellabs Pty Ltd, Brookvale NSW, Australia). The kit is based on the indirect immunosorbent assay (ELISA) principle and designed for detection of malaria specific IgG antibody response in serum against four human malaria species (*Plasmodium falciparum*,* P. vivax*,* P. malariae,* and* P. ovale*). This kit has been independently validated against other diagnostic methods with (95.5% sensitivity and 92.2% specificity) [9]. ELISA microwells are coated with a panel of recombinant malaria antigens. Serum samples are diluted at 1/100 and incubated in the microwells. After incubation and washing, the wells are incubated with a conjugate (mouse anti-human monoclonal antibodies, labeled with the horse radish peroxidase enzyme). After washing the wells, a chromogen substrate (tetramethylbenzidine) is added and incubated, leading to color development which is proportional to the amount of malaria antibodies present in the sample. Negative and positive serum controls are supplied in the kit. A cutoff of OD = 0.150 was developed in our laboratory by using mean (OD = 0.059) + 3SD (SD = 0.0267 × 3) readings of negative samples. Negative samples were derived from individuals not exposed to malaria based on travel history. Test samples were evaluated against this cutoff level as positive or negative for malaria antibodies. *F*-test and Pearson coefficient tests were used to assess any significance of antibody positivity between 10 centers of blood collection.

### 2.4. Immunoassay for Malaria Antigens

Histidine rich proteins specific for* P. falciparum* were detected by using an ELISA kit (Malaria Antigen CELISA, Cellabs Pty Ltd, Brookvale NSW, Australia) [10, 11]_._ Serum samples diluted 1/2 in PBS/T were tested in the ELISA wells coated with capture monoclonal antibodies. It is known that detectable levels of* P. falciparum* specific HRP antigens are present in serum [11]. Histidine protein antigens present in the blood sample were sandwiched by the enzyme labeled detector monoclonal antibodies. The amount of sandwiched histidine proteins was proportional to the color intensity following the addition of a chromogen substrate into the ELISA wells. The plates were read in a dual wavelength (450/620 nm) plate reader. The OD readings of test samples were recorded and compared to the kit positive (contained natural histidine rich proteins released by* P. falciparum* parasites) and negative (media blank) controls. Any readings above the cutoff level OD > 0.12 (mean + 3SD of negative samples) were classified as positive.

### 2.5. Immunoassay for Plasmodium Specific Lactate Dehydrogenase

A second line of testing was performed for circulating plasmodium genus-specific lactate dehydrogenase (Pan-pLDH-ELISA) antigens in serum samples [12]. Presence of Pan-pLDH antigens in serum samples is indicative of active malaria infection. The pLDH CELISA kit [(Cellabs Pty Ltd, Brookvale NSW, Australia with In-house specificity (97%) and sensitivity (95%))] assay is based on the capture of plasmodium-specific lactate dehydrogenase antigens in serum samples. The kit positive and negative controls provided a cutoff (mean + 3SD) of OD = 0.100. Any sample reacting higher than the cutoff was considered positive. The Pan-pLDH positive samples are suggestive of prevailing active malaria carriers in the community.

## 3. Results

### 3.1. Plan of Study

**Figure 2:**
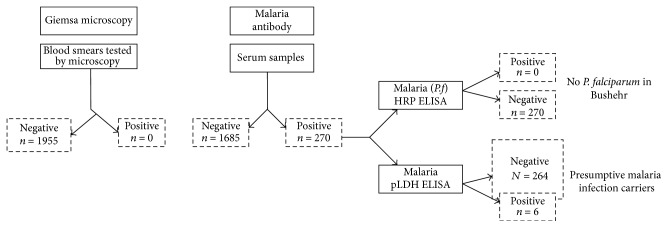
Flow chart and overall results.

The flow chart (Figure 2) details the testing procedure followed and overall results obtained. Initially blood smear microscopy was used for identifying active malaria cases. Both* P. falciparum* and* P. vivax* blood smear positive slides and published blood stage photographs of* P*.* malariae* and* P. ovale* were used as reference while examining the stained smears prepared from a total of 1955 blood samples. No active malaria cases were identified based on blood smear microscopy. Then we tested the serum samples in a validated malaria antibody detection ELISA identifying sero-positive individuals and malaria antigen ELISAs were used for identifying individuals carrying malaria. Results of microscopy, antibody, and antigen levels that were found in 10 different centers are documented in Table 1. No significant difference was seen in the antibody positivity between 10 different centers (Table 1) (*F*-test and Pearson coefficient test).

### 3.2. Participants

Of the 1955 individuals enrolled for the study, a total of 1085 (55.5%) were men and 870 (44.5%) were women. The mean age was 32.82 ± 14.61 years. There were 1422 local residents and 533 travelers. About 5 mL blood samples were collected from each volunteer by venipuncture, and the whole blood was stored at 4C in the refrigerator until use in the assays. Serum samples were frozen at −20C until use. Travelers were also included in this study for testing the possibility of them bringing active malaria infection from endemic into nonendemic regions. All samples from local residents as well as from travelers were tested under identical condition for prevailing malaria antibodies as well as for malaria antigens.

### 3.3. Testing for Malaria Antibodies and Antigens

Pan Malaria IgG antibody test was employed. The kit positive control reacted fairly strongly (mean OD = 2.581) which is 44x higher than the OD reading of the negative control. This showed that Pan Malaria antibody kit possesses a high level (>40x) of discrimination between negative and positive samples. A battery of in-house negative samples plus kit negative control samples were initially tested and a cutoff (mean + 3SD) (OD = 0.150) was determined. This cutoff served as the basis for differentiating positive samples in malaria antibody assay. In a similar manner we also developed a cutoff level for malaria antigen ELISAs. Data in Table 1 shows that out of 1955 blood samples tested 270 samples reacted for malaria antibodies and 6 samples reacted for malaria antigens.

### 3.4. Malaria Antibody Assay-Frequency Distribution of ELISA Absorbance Value

**Figure 3:**
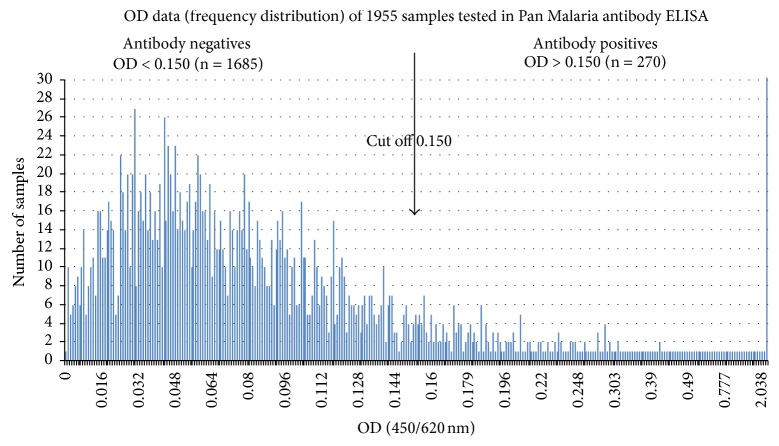
Frequency distribution of OD readings of 1955 serum samples tested at 1/100 dilution in Pan Malaria IgG CELISA. The samples (*n* = 270) showing OD readings >0.150 are regarded as positive.

**Figure 4:**
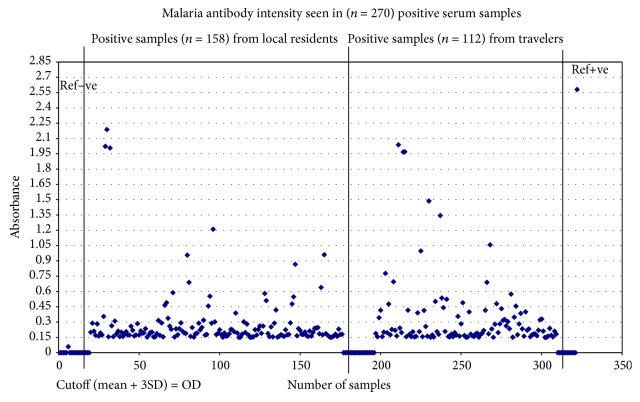
Scattergram of 270 malaria antibody positive samples OD = 0.15.

Out of a total of 1955 samples tested in Pan Malaria IgG antibody CELISA (see frequency distribution of OD readings in Figure 3), 270 samples (13.8%) reacted positively for malaria antibodies. A cutoff (mean + 3SD) OD = 0.150 was used to identify positively reacted samples.  Figure 4 shows intensity of OD readings above the cutoff; 11.1% of local resident samples and 21% of traveler samples reacted positively in the antibody assay (Figure 4). Bushehr region therefore contained local residents with malaria antibodies which are suggestive of their exposure to malaria infection. The origin of their malaria exposure is beyond the scope of this investigation. The premise that travelers were the cause of local infection in Bushehr was further investigated by using plasmodium antigen specific assays.

### 3.5. Exposure to Malaria Significantly Influenced by Age

Data based on age are shown in Table 2; highest rates of seropositivity were seen in two age groups (i.e., 21–30 and 31–40).

### 3.6. Testing for* P. falciparum* Cases

Malaria antibody positive serum samples were tested for presence of* P. falciparum* specific histidine rich protein antigens in serum samples by using a commercial kit proven to be highly sensitive for detecting* P. falciparum* cases [10, 11]. None of the antibody positive samples tested were found to be positive (cutoff OD = 0.12) for* P. falciparum* antigen. This showed that no* P. falciparum* carriers were present in the malaria antibody positive samples based on histidine rich protein ELISA [10].

### 3.7. Pan-pLDH Assay for Identification of Active Malaria Carriers

**Figure 5:**
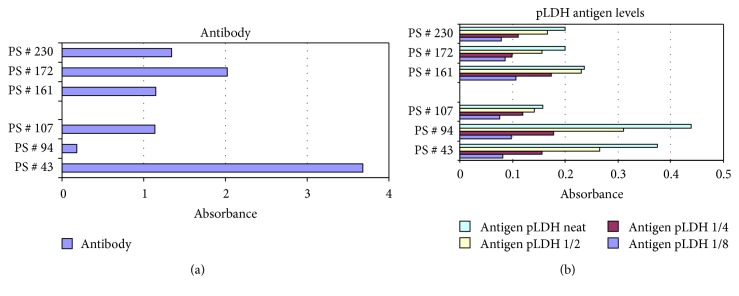
Comparative levels of malaria antibody and pLDH antigens in six samples.

Next, a total of 270 antibody positive samples were further tested in plasmodium specific lactate dehydrogenase ELISA test (Pan-pLDH assay cut off (mean + 3SD; OD = 0.10); 6 antibody positive samples (PS) (PS numbers 43, 94, and 107 from residents samples and PS numbers 161, 172, and 230 from travelers samples) reacted positively in pLDH antigen assay (Figure 5). When we referred back to their malaria antibody levels, PS numbers 43 and 172 showed elevated antibody readings, respectively (OD = 3.679, 2.023) and the latter also showed elevated pLDH level. The three pLDH positive samples were derived from the travelers from endemic Sistan (PS number 161) and Hormozgan (PS numbers 172 and 230) provinces. Blood smears from six Pan-PLDH antigen positives were reexamined microscopically and none were positive.

## 4. Discussion and Conclusions

The national malaria control programs initiated in Iran during the 1950's resulted by 1977 [2–4] in the eradication of infection from the Caspian Sea region in the north and in the south, a substantial reduction in transmission in the coastal plains along the Persian Gulf. Malaria was then restricted to the southeastern provinces of Hormozga, Sistan, and Baluchistan and the tropical region of Kerman. However, due to displacement of people from neighboring countries, malaria reemerged in Iran [13]. Recent review on endemic infectious diseases in Afghanistan points towards possible impact of malaria in adjacent countries and that impact may occur through a guest worker (traveller) coming into Iran [14]. The Bushehr region, being in the southwest, is out of the declared endemic south east regions. Our investigation mainly focused in locating possible active carriers, if any, in the hypoendemic Bushehr region. Our main purpose was to identify individuals potentially exposed to malaria, as they may harbour infection and become a source for transmission within a region. Our purpose was first to identify individuals showing malaria antibodies in serum as an evidence for exposure. Then samples from those malaria antibody positive individuals were further tested for circulating malaria antigens by antigen capture ELISA [15]. The lack of positive blood smears amongst a total of 1955 blood samples tested by malaria microscopy indicated no evidence for clinical malaria cases in the Bushehr community.

We subsequently focused our investigation towards identifying those individuals exposed to malaria. It is known that antibodies prevail in the circulation for many years after exposure to malaria infection [6]. It is also known that the presence of malaria specific antibody in the serum is helpful in determining if a particular individual has been exposed to the malaria or not. Out of 1955 serum samples tested, 270 samples were found to be positive (OD > 0.150) for malaria specific antibodies. 21% of traveler samples reacted positively for antibodies as opposed to 11% of local residents samples. Based on our Giemsa methodology none were showing active malaria infection. We recorded relatively higher percentage of antibody response in certain age groups (21 to 40) (Table 2). This antibody positivity is based upon the sensitivity and specificity of the assay we employed. The Pan Antibody positivity was determined by the Pan Malaria Antibody assay, an assay that has been independently shown to have high specificity and sensitivity [9], so we believe our results to be reliable and acceptable. It suggests the current situation prevailing in the community. On the whole, only a small proportion of the general population was found to be positive for malaria antibody response. However, a large part of the study population (86%) did not have any indication of seropositivity. We therefore conclude that malaria in Bushehr region is not alarming. In support of this statement, we did not find any evidence for* P. falciparum* active infection amongst the exposed group of 270 samples, with sensitive and well validated histidine rich proteins ELISA tests that have been used with field malaria samples [10, 11]. Utility of histidine rich proteins and parasite lactate dehydrogenase tests is well documented [10–12].

We further tested those malaria antibody positives with the pLDH CELISA kit to detect plasmodium specific lactate dehydrogenase enzyme. Six individuals were found to be positive at a very low level of pLDH. Because the* P. falciparum* specific histidine rich proteins ELISA results were consistently negative, it is reasonable to assume that these six individuals may have been exposed to non-falciparum malaria species.

A point of explanation is needed on our results. Here our observation has shown only the “exposure” situation and not as any degree of “infection.” It is assumed that Bushehr community is naive to malaria. It is postulated that malaria is introduced into Bushehr by a traveller from an adjacent malaria endemic area, and that results in local transmission. Our findings substantiate this situation very well as Giemsa-based blood smear microscopy was consistently negative and no active malaria case was observed. Results can be attributed only as “exposure” situation but not as “infection” situation in Bushehr. If we see overall malaria antigen results, it is clearly apparent that absence of histidine proteins shows there is no active infection prevailing. However in absence of histidine proteins, occurrence of pLDH in some of the individuals may show that they may carry some sub-microscopic stages of P. vivax invariably in the form of dormant hypnozoites [16, 17]. Hypnozoites persists for a long period of time and may be source of additional infections in the hypo-endemic sites. The role of hypnozoites as dormant parasites is very well established in US war veterans. This may be the case in Bushehr where no active infections shown but pLDH indicated the possibility of sub-microscopic infection.

Because we restricted antigen testing only to antibody positive samples, a question may be raised on the possibility of antibody negative samples showing some level of antigens. This criticism can be answered by stating that, if malaria is endemic in the community, then such antigen positive individuals should have tested positive for malaria antibodies, because malaria antibodies develop soon after infection. It is unlikely that antibody negative individuals would be positive for malaria antigens. We, therefore, conclude that our approach of only testing for malaria antigen in antibody positive individuals is appropriate.

Recently* P. vivax* and* P. falciparum* have been reported from central Iran, aside from known records of infection from southeastern endemic regions [18, 19]. There is also a clear indication of two prevailing species (*P. vivax *in 94% cases and* P. falciparum *in 6% cases) in Iran. Despite a recent report of a malaria slide positivity rate of 1.19% in Iran [19], we did not record a single case of infection by blood smear microscopy in the Bushehr province. However, we should point out that our conclusions are based on the results that came out of the immunological assays we have employed during this study. It is quite possible that inclusion of other tools such as polymerase chain reaction may be useful in generating additional information. This is planned for future studies.

Our work has shown the usefulness of immunoassays in identifying individuals exposed to malaria and also in detecting individuals carrying potentially active infections albeit at a possibly low level parasitaemia that are not detectable by Giemsa microscopy. Asymptomatic malaria in hypoendemic areas is very important [20, 21]. This strategy of testing we followed here at Bushehr may be useful for indentifying asymptomatic malaria exposure in 34 malaria-eliminating countries.

## Figures and Tables

**Table 1:** Overall results of Pan Malaria antibody and pLDH antigen ELISA assays performed on blood samples obtained from 1955 participants in Bushehr, Iran.

Area^1^	Number tested^2^	Antibody positive	pLDH antigen positive
Assaloyeh Center	221 (11.30%)	37 (16.74%)	2 (0.90%)
Chah Mobarak Center	187 (9.56%)	39 (20.85%)	1 (0.53%)
Bandare Taheri Center	204 (10.43%)	15 (7.35%)	1 (0.49%)
Nakhle Taghi Center	105 (5.37%)	17 (16.19%)	1 (0.95%)
Ghods Center	366 (18.72%)	65 (17.76%)	1 (0.27%)
17 Shahrivar Center	152 (7.77%)	12 (7.90%)	0.0 (0.0%)
Banak Center	112 (5.72%)	17 (15.18%)	0.0 (0.0%)
PSEEZ-Center1	187 (9.56%)	31 (16.58%)	0.0 (0.0%)
PSEEZ-Center2	331 (16.93%)	24 (7.25%)	0.0 (0.0%)
PSEEZ-Center3	90 (4.60%)	13 (14.44%)	0.0 (0.0%)

Total	1955 (100%)	270 (13.81%)	6 (0.30%)

^1^Statistical analyses revealed no significant differences in antibody positivity between different centers (*F*-Test NS) and Pearson coefficient (0.723 NS).

^2^Microscopy indicated no positive results in the samples tested from 10 centres and also no sample was found to be positive for *P. falciparum* specific antigens.

**Table 2:** The range of age groups of participants and overall percentages of antibody positives seen when serum samples were tested in Pan Malaria antibody ELISA.

Age (range) of participants	Number of antibody positives/total number tested	Age group percentage (positivity)	Overall percentage (positivity)
<11 years	3/38	7.9%	0.15%
11–20	20/216	9.2%	1%
21–30	116/670	17.3%	5.93%
31–40	92/583	15.8%	4.7%
41–50	18/264	6.8%	0.92%
51–60	7/105	6.7%	0.36%
>61	14/79	17.7%	0.72%

Total (overall percentage)	270/1955		13.8%
